# Dual role of interferon-gamma in the response of melanoma patients to immunotherapy with immune checkpoint inhibitors

**DOI:** 10.1186/s12943-025-02294-x

**Published:** 2025-03-20

**Authors:** Piotr Wawrzyniak, Mariusz L. Hartman

**Affiliations:** Department of Molecular Biology of Cancer, Medical University of Lodz, 6/8 Mazowiecka Street, 92-215 Lodz, Poland

**Keywords:** CTLA-4, Immune checkpoint inhibitors (ICIs), Interferon-gamma (IFN-γ), LAG-3, Melanoma, PD-1, PD-L1

## Abstract

Interferon-gamma (IFN-γ) is a cytokine produced mainly by immune cells and can affect cancer cells by modulating the activity of multiple signaling pathways, including the canonical Janus-activated kinase/signal transducer and activator of transcription (JAK/STAT) cascade. In melanoma, IFN-γ can exert both anticancer effects associated with cell-cycle arrest and cell death induction and protumorigenic activity related to immune evasion leading to melanoma progression. Notably, IFN-γ plays a crucial role in the response of melanoma patients to immunotherapy with immune checkpoint inhibitors (ICIs), which are currently used in the clinic. As these agents target programmed death-1 (PD-1) and its ligand (PD-L1), cytotoxic T-lymphocyte-associated protein-4 (CTLA-4) and lymphocyte-activation gene 3 (LAG-3), they are designed to restore the antimelanoma immune response. In this respect, IFN-γ produced by cells in the tumor microenvironment in response to ICIs has a beneficial influence on both immune and melanoma cells by increasing antigen presentation, recruiting additional T-cells to the tumor site, and inducing direct antiproliferative effects and apoptosis in melanoma cells. Therefore, IFN-γ itself and IFN-γ-related gene signatures during the response to ICIs can constitute biomarkers or predictors of the clinical outcome of melanoma patients treated with ICIs. However, owing to its multifaceted roles, IFN-γ can also contribute to developing mechanisms associated with the acquisition of resistance to ICIs. These mechanisms can be associated with either decreased IFN-γ levels in the tumor microenvironment or diminished responsiveness to IFN-γ due to changes in the melanoma phenotypes associated with affected activity of other signaling pathways or genetic alterations e.g., in *JAK*, which restricts the ability of melanoma cells to respond to IFN-γ. In this respect, the influence of IFN-γ on melanoma-specific regulators of the dynamic plasticity of the cell phenotype, including microphthalmia-associated transcription factor (MITF) and nerve growth factor receptor (NGFR)/CD271 can affect the clinical efficacy of ICIs. This review comprehensively discusses the role of IFN-γ in the response of melanoma patients to ICIs with respect to its positive influence and role in IFN-γ-related mechanisms of resistance to ICIs as well as the potential use of predictive markers on the basis of IFN-γ levels and signatures of IFN-γ-dependent genes.

## Background

**Fig. 1:**
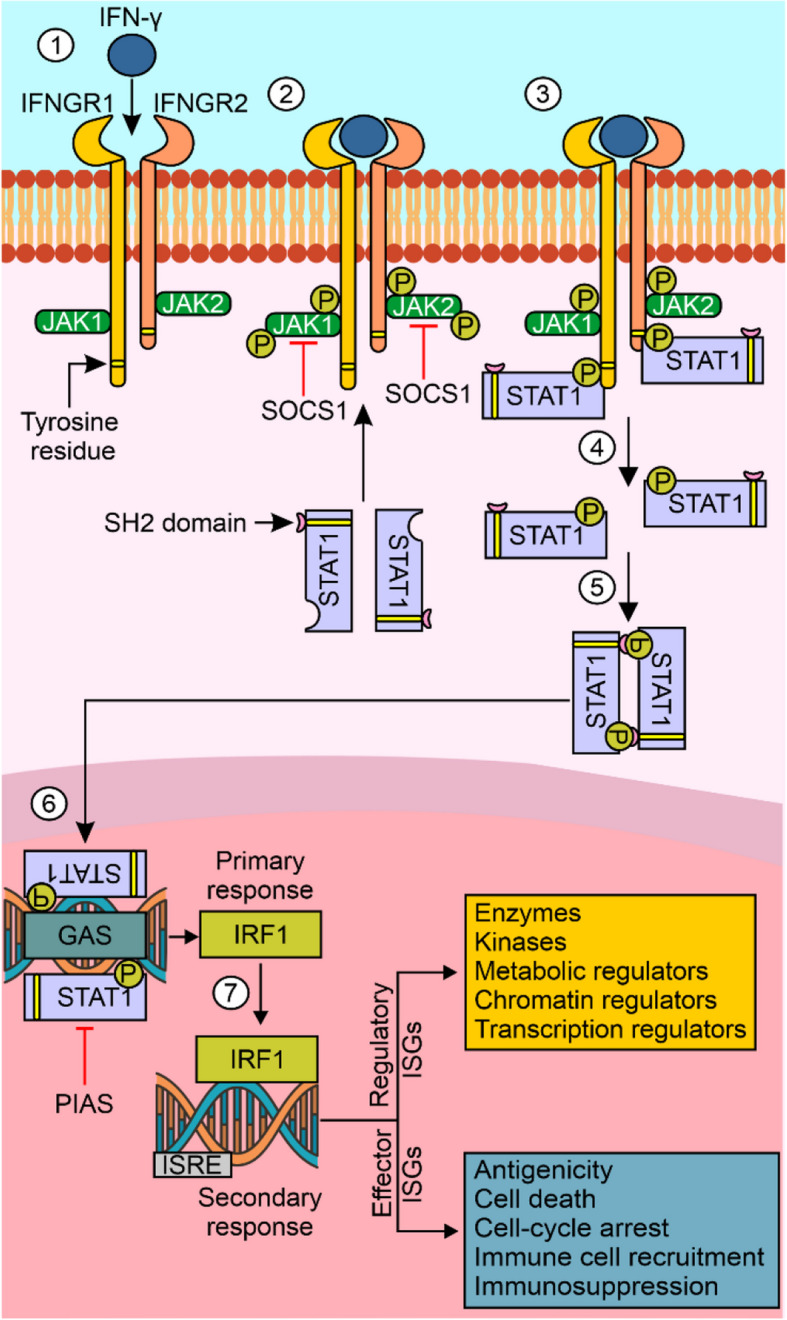
Interferon-gamma (IFN-γ) signaling is canonically mediated through the JAK/STAT pathway. (1) IFN-γ binds with interferon-gamma receptor 1 (IFNGR1) and 2 (IFNGR2). (2) The ligand-receptor connection causes Janus-activated kinase 1 and 2 (JAK1/2) activation via transphosphorylation, and the tyrosine residues are phosphorylated to form a signal transducer and activator of transcription 1 (STAT1) docking site. Suppressor of cytokine signaling 1 (SOCS1), a negative regulator of this step, can inhibit the phosphorylation of JAK1 and JAK2. (3) STAT1 is phosphorylated at the docking site. (4) Phosphorylated STAT1 is detached from the receptor. (5) STAT1 dimerizes via SH2-domain-phosphotyrosine interactions. (6) STAT1 dimers are transported to the nucleus and bind to DNA at the interferon-gamma activated site (GAS), which can be inhibited by the protein inhibitor of activated STAT (PIAS). (7) The primary transcriptional response involves the induction of interferon regulatory factor 1 (IRF1), which is followed by a secondary response after the binding of IRF1 to interferon-stimulated response elements (ISREs). IRF1 bound to ISRE promotes the expression of interferon-stimulated genes (ISGs)

Under physiological conditions, interferon-gamma (IFN-γ) is produced predominantly by T-cells, B-cells, natural killer (NK) cells, and natural killer T (NKT) cells in response to a variety of stimuli, including interleukin (IL)-12, IL-15, IL-18, type I interferons and pathogen-associated molecular patterns (PAMPs) [1]. The main effector role of the IFN-γ homodimer is driven through interferon-gamma receptor (IFNGR), which consists of two subunits: IFNGR1 (α subunit) and IFNGR2 (β subunit). Receptor dimerization is a tightly ligand-driven process (Fig. 1) [2]. While IFNGR1 is constitutively expressed in all nucleated cells, the level of IFNGR2 is strictly controlled. As IFNGRs lack kinase activity, they are associated with the nonreceptor Janus kinases JAK1 and JAK2. After the ligation of IFN-γ and receptor activation, autophosphorylation of JAK2 is triggered at Tyr^1007^/Tyr^1008^ following the transphosphorylation of JAK1 at Tyr^1022^/Tyr^1023^, which leads to the activation and nuclear translocation of signal transducer and activator of transcription 1 (STAT1) and 3 (STAT3) to bind to the interferon-gamma activated site (GAS) elements of IFN-γ-stimulated genes (ISGs), including interferon regulatory factor 1 (IRF1) (Fig. 1). IRF1 was shown to coordinate the expression of genes involved in the secondary response to IFN-γ [3]. The activity of the JAK/STAT pathway is regulated by negative feedback by suppressor of cytokine signaling 1 (SOCS1) and 3 (SOCS3) proteins, which form complexes with JAK1/2, whereas protein inhibitor of activated STAT (PIAS) suppresses the transcriptional activity of STAT dimers (Fig. 1) [4]. In melanoma, IFN-γ can induce tryptophan‐tRNA ligase (WARS), which is associated with STAT1, limiting the activity of IFN-γ/JAK/STAT signaling [5]. IFN-γ-dependent activation of the JAK/STAT/IRF1 signaling pathway can induce cytotoxic and cytostatic effects in cancer cells [6], although it was shown that only a fraction of melanoma cell lines can respond to IFN-γ with potent induction of its typical targets [7]. A moderate proapoptotic potential of IFN-γ was demonstrated in melanoma cells, while downregulation of IFNGR1 abolished IFN-γ-mediated cell death [8]. Mechanistically, IFN-γ-dependent apoptosis relies on increased extracellular signal-regulated kinase (ERK) activity, which induces a stress response leading to cell death associated with the activity of death receptor 5 (DR5) and NOXA encoded by phorbol-12-myristate-13-acetate-induced protein 1 (*PMAIP1*) [9]. In turn, IFN-γ induces cell cycle arrest in melanoma cells via the upregulation of JAK/STAT/miR-29 signaling, which is followed by the inhibition of cyclin-dependent kinase 6 (CDK6) [10], the accumulation of the cyclin-dependent kinase inhibitor p27 and the downregulation of S-phase kinase-associated protein 2 (SKP2) [11]. IFN-γ suppresses melanoma tumor growth [12, 13] by inducing ubiquitin-specific peptidase 18 (USP18) and sensitizing tumor cells to immunosurveillance [12], which may be limited by tumor-associated macrophages (TAMs) [14].

As all types of cancer cells, melanoma cells can largely avoid host immune checkpoints and surveillance, and IFN-γ contributes to these features, thereby exerting protumorigenic effects. IFN-γ promotes ultraviolet-mediated melanomagenesis [15] and melanoma cell survival by regulating CD74-macrophage-migration inhibitory factor (MIF) signaling, leading to the activation of AKT phosphorylation and the expression of IL-6, IL-8, and B-cell leukemia/lymphoma-2 (BCL-2) [16]. In turn, the secretion of soluble CD74 (sCD74) was observed in melanoma cells and macrophages, while sCD74 suppressed melanoma cell growth and induced apoptosis, inhibiting the MIF/CD74/AKT pathway under IFN-γ stimulation [17]. By downregulating the NKG2D ligand crucial for the NKG2D-mediated response of NK cells, IFN-γ facilitates immune escape of major histocompatibility complex-I (MHC-I)-negative melanoma cells [18]. IFN-γ promotes tumorigenicity and metastasis in a syngeneic mouse model of melanoma, and this effect is STAT1-dependent and associated with the influence of IFN-γ on γδ T-cells [19]. IFN-γ contributes to melanoma progression, which can be suppressed by targeting neuronal nitric oxide synthase (nNOS) [20]. The contribution of IFN-γ to the metabolic reprogramming of melanoma cells to promote immune evasion has also been demonstrated [21]. IFN-γ increases the expression of nicotinamide phosphoribosyltransferase (*NAMPT*), an enzyme involved in NAD^+^ biogenesis, which is associated with the metabolic reprogramming of melanoma cells and increased melanoma growth in vivo [22]. Sustained low levels of IFN-γ increase the expression of molecules involved in tumor immune evasion, including programmed death-1 ligand 1 (PD-L1) and 2 (PD-L2) and cytotoxic T-lymphocyte-associated protein-4 (CTLA-4) [23]. In this respect, the preferential contribution of JAK1 over JAK2 to the induction of PD-L1 by IFN-γ in melanoma cells has been reported [24]. IFN-γ can regulate the kynurenine pathway, which is activated by indoleamine 2,3-dioxygenase 1 (IDO1) to catabolize tryptophan into kynurenine and exert immunosuppressive effects [25] in both melanoma and normal cells in the skin [26]. Increased expression of immunosuppression-related genes, including *IDO1*, was detected after downregulation of *WARS* without a change in the phosphorylation status of STAT1 [5], whereas a strong correlation between the levels of IFN-γ and IDO-1 was found in different types of melanoma [27, 28].

While IFN-γ has a multifaceted role in melanoma, IFN-γ was also found to regulate immune responses upon immunotherapy with immune checkpoint inhibitors (ICIs) [29]. This review focuses on both the beneficial effects of IFN-γ on the response of melanoma patients to ICIs and the contribution of IFN-γ to ICI resistance.

### A short overview of the mechanisms of immunotherapy with ICIs

**Fig. 2:**
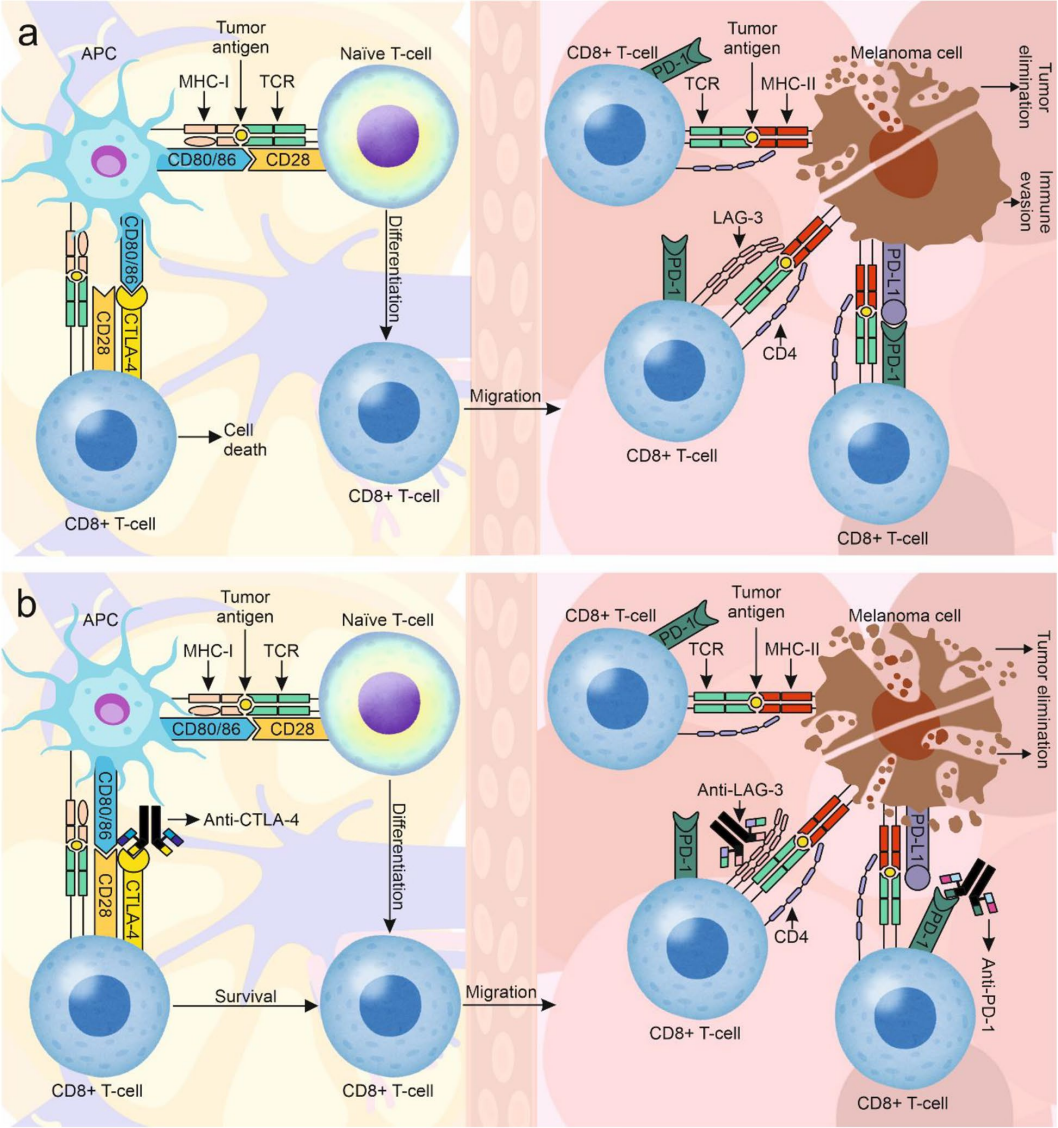
Mechanisms of action of immune checkpoints and inhibitors of immune checkpoints (ICIs).** A** In lymph nodes, naïve T-cells are differentiated by interactions with antigen-presenting cells (APCs), which present melanoma antigens by binding major histocompatibility complex-I (MHC-I) to the T-cell receptor (TCR) and delivering a costimulatory signal through the interaction of CD80/86 with CD28. Upon activation, CD8 + T-cells express the cytotoxic T-lymphocyte-associated protein-4 (CTLA-4) receptor, which competes with CD28 to bind CD80/86, thereby regulating T-cell activity and protecting tissues from excessive immune responses. Activated CD8 + T-cells migrate to the tumor microenvironment, where they recognize tumor antigens via the binding of TCRs to MHC-II molecules on the surface of melanoma cells. A secondary costimulatory signal is provided by CD4 receptors on activated T-cells, which interact with MHC-II. However, melanoma cells can evade immune responses by fostering an immunosuppressive microenvironment e.g., by expressing programmed death-1 ligand 1 (PD-L1), a ligand for the programmed death-1 (PD-1) immune checkpoint. Another example is the upregulation of lymphocyte-activation gene 3 (LAG-3) on activated T-cells, which serves as another immune checkpoint. The activation of these checkpoints in the tumor microenvironment leads to immune escape in melanoma, as it impairs the effector function of CD8 + T-cells. **B** Mechanisms of action of immune checkpoint inhibitors. The use of anti-CTLA-4 antibodies increases the pool of active CD8 + T-cells, thereby increasing immune system reactivity. In turn, anti-PD-1 and anti-LAG-3 antibodies help sustain the effector function of active CD8 + T-cells, leading to efficient melanoma cell death by maintaining an unfavorable microenvironment for the tumor

Currently approved immunotherapies with ICIs for patients with melanoma include the following antibodies: (1) nivolumab and pembrolizumab against programmed death-1 (PD-1) and atezolizumab against its ligand (PD-L1), (2) ipilimumab against CTLA-4, and (3) relatlimab against lymphocyte-activation gene 3 (LAG-3), which are used as monotherapies or in specific combinations, also with other classes of antimelanoma therapies [30]. Currently used ICIs are generally designed to maintain the effector function of T-cells in the tumor microenvironment by inhibiting their premature silencing caused directly and indirectly by cancer cells (Fig. 2) [30]. Naïve T-cells need at least two signals to induce activation. The first signal is through the binding of antigen-presenting cells (APCs) by MHC-I or MHC-II to recognize the tumor antigen with the T-cell receptor (TCR) [31]. The second costimulatory signal is triggered by the binding of CD80 (B7-1) or CD86 (B7-2) molecules on APCs with CD28 on T-cells. Once an appropriate level of binding is reached, IL-2 is secreted to activate and differentiate T-cells [31–33]. CTLA-4 is a type 1 transmembrane glycoprotein of the immunoglobin superfamily with a structure similar to that of CD28. *CTLA4* is located on chromosome 2 and consists of 4 exons encoding a leading peptide (exon 1), a CD80/CD86-binding site and a dimerization site (exon 2), a transmembrane region (exon 3), and a cytoplasmic tail (exon 4). In humans, CTLA-4 has two isoforms: a full-length isoform containing all exons and a soluble isoform lacking exon 3 present in the serum. The expression of CTLA-4 is induced by T-cell activation and can lead to T-cell death through competing binding of CTLA-4 with CD28 for CD80/CD86, which downregulates T-cells [32–34]. Compared with CD28, CTLA-4 is able to bind to CD80/CD86 receptors with greater affinity [33, 34]. There are several additional inhibitory immune checkpoints that limit the antitumor response of activated T-cells within the tumor microenvironment, including the transmembrane glycoprotein PD-1 [34] and LAG-3 [35]. Inhibition of activated T-cell immunity requires two signals: the first signal is triggered by binding PD-1 to PD-L1 or PD-L2 followed by phosphorylation of tyrosine residues in the immunoreceptor tyrosine-based inhibitory motif (ITIM) and immunoreceptor tyrosine-based switch motif (ITSM) domains in the cytoplasmic region of PD-1, and the second signal is triggered by binding the TCR with MHC-II [36]. PD-L1 and PD-L2 are expressed on many cell types, including tumor cells [36]. The LAG-3 receptor structurally resembles the CD4 molecule, and LAG-3 homodimerization and glycosylation are crucial for its binding to MHC-II [37]. Despite a greater affinity for MHC-II than for CD4, LAG-3 does not compete for the binding site within CD4 but interacts with another part of MHC-II [38]. Considering these mechanistic bases, the anti-PD-1, anti-CTLA-4 and anti-LAG-3 antibodies used in the clinic either potentiate the physiological immune response or reconstitute functional antitumor immunity. The use of anti-PD-1 and anti-CTLA-4/anti-LAG-3 ICIs in combination enables synergistic activity on the basis of distinct spatiotemporal actions during the response of the immune system. While CTLA-4 is responsible for inhibiting the activation of naïve T-cells in the lymph node during the initial immune response, PD-1/PD-L1 and LAG-3 play significant roles in the tumor microenvironment [39].

### Beneficial role of IFN-γ in the response of melanoma patients to ICIs

**Fig. 3:**
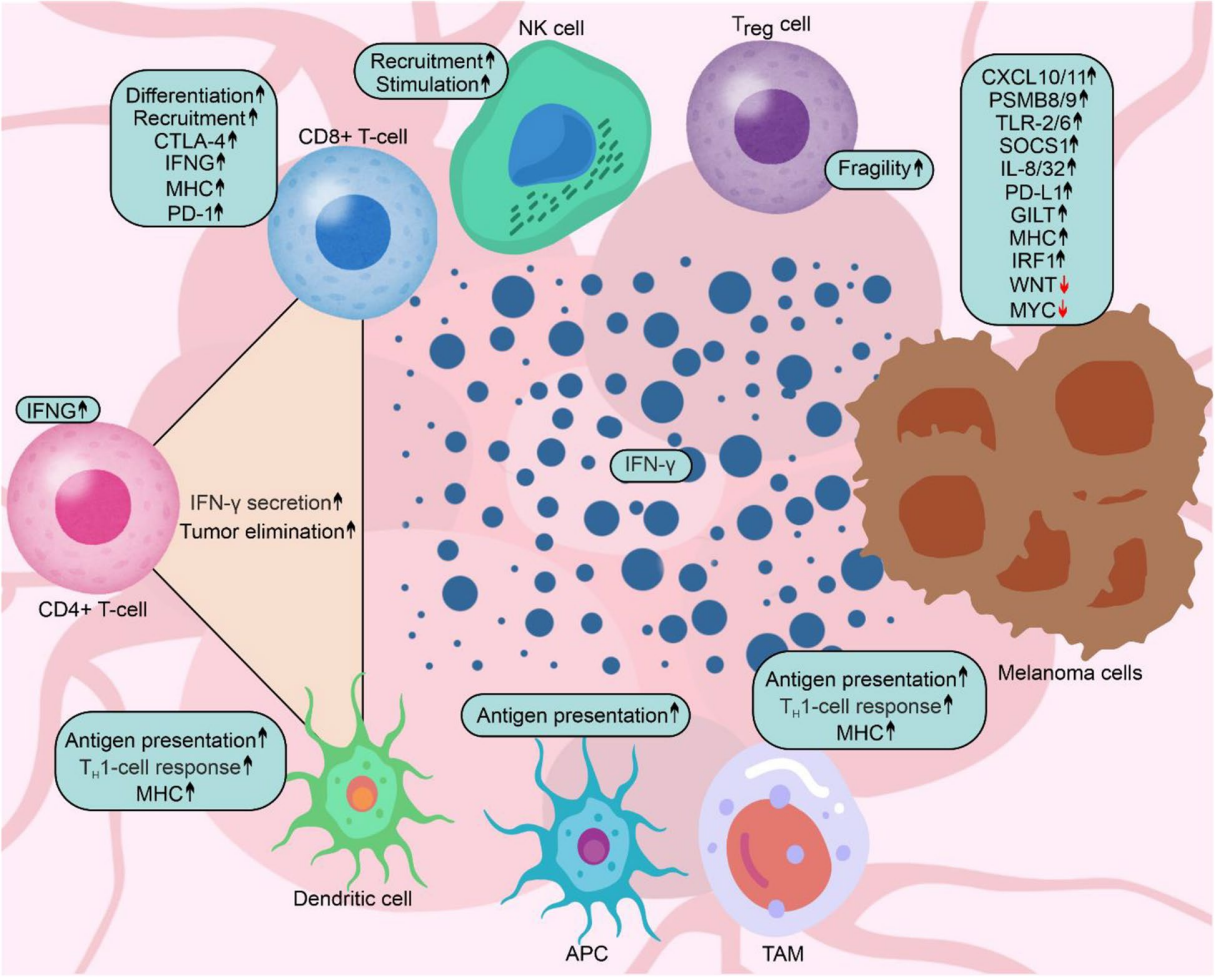
The beneficial effects of IFN-γ for melanoma patients treated with ICIs. IFN-γ produced by immune cells influences other immune cells to support the anticancer response during immunotherapy. In this respect, IFN-γ is a part of the functional triad between CD4 + T-cells, CD8 + T-cells, and dendritic cells. In addition, IFN-γ influences melanoma cells by affecting the expression of genes encoding proteins involved in the immune response. A detailed description of these mechanisms is included in the text

The response to immunotherapy is strongly dependent on the microenvironment of the tumor, including tumor-infiltrating immune cells, and the tumor immunotypes that reciprocally regulate immune cell responses [40, 41]. In this respect, "hot" and "cold" tumors can be distinguished. “Hot” tumors are characterized by high T-cell infiltration; high expression of CTLA-4, LAG-3, T-cell immunoglobulin domain and mucin domain‐3 (TIM-3) and PD-1; and high inflammation. Collectively, these features support the immune response, whereas "cold" tumors are characterized by a weak immune response [42]. The tumor microenvironment dynamically changes during immunotherapy [40]. Increasing fractions of PD-1^high^/CTLA-4^high^ tumor-infiltrating CD8 + T-cells are correlated with the response to therapy and progression-free survival (PFS) in patients with melanoma [43]. In this respect, a study by Bagaev et al. [44] comprehensively divided melanomas into four categories on the basis of functional gene expression in the tumor microenvironment. Melanomas are classified as immune-inflamed (either fibrotic or nonfibrotic) or immune-desert (either fibrotic or depleted). Notably, patients with immune-inflamed melanomas generally exhibit greater overall survival (OS) and PFS when receiving anti-PD-1 or anti-CTLA-4 immunotherapy than those with melanomas categorized into the immune-desert type [44]. Other classifications based on the characteristics of the melanoma microenvironment with respect to tumor immunity have been shown, revealing the crucial role of IFN-γ in antimelanoma immunity [45, 46]. In this respect, IFN-γ produced by cells in the tumor microenvironment in response to ICIs has a substantial influence on both immune and melanoma cells, leading to beneficial effects supporting the overall response to immunotherapy by increasing MHC expression/antigen presentation, recruiting additional T-cells to the tumor, and inducing direct antiproliferative effects and apoptosis in melanoma cells (Fig. 3). Inhibition of PD-1 increases the IFN-γ level at the tumor site, which is followed by enhanced chemokine-dependent trafficking of immune cells into melanoma tumors [47]. *IFNG*, encoding IFN-γ, was the only common gene upregulated in the T-cells of immunotherapy-receiving patients classified in all three cohorts as either being treated with anti-PD-1, anti-CTLA-4 or combination therapy [48]. IFN-γ induces regulatory T-cell (T_reg_) fragility, which is required for an efficient response to anti-PD1 therapy in melanoma [49]. In response to ICIs, IFN-γ modulates inflammatory and immune responses by affecting TAMs and dendritic cells (DCs) via increasing *MHC* expression and antigen presentation, and promoting T-helper 1 (T_H_1)-cell response, although feedback mechanisms attenuating antitumor immunity are also involved [50]. Accordingly, ICI-responding melanoma patients are characterized by higher basal levels of T-cells than ICI-resistant patients [51, 52]. Increased secretion of IFN-γ as a part of the functional triad between CD4 + T-cells, CD8 + T-cells and dendritic cells was shown to determine the effective response to ICIs in melanoma through sufficient reprogramming of CD8 + T-cells [53]. It has also been demonstrated that a combination of anti-LAG-3 and anti-PD-1 therapy stimulates NK cells, which is associated with an increase in the IFN-γ response particularly in patients who exhibit an objective complete or partial response [54]. Recently, CD8 + T-cells deficient in both PD-1 and LAG-3 were shown to exhibit enhanced tumor clearance in a mouse model of melanoma due to increased secretion of IFN-γ [55], and a combination of relatlimab and nivolumab affected the differentiation of CD8 + T-cells by enhancing responses to TCR receptor and IFN-γ signaling, which was associated with enhanced effector functions of T-cells [56].

In addition to its influence on immune cells during treatment with ICIs, IFN-γ largely affects the response to immunotherapies by modulating multiple processes in melanoma cells. Melanoma cells are characterized by higher levels of IFNGRs than of IFN receptor type I, which explains why they are more sensitive to IFN-γ than other types of interferon. The responsiveness of melanoma cells to IFN-γ is associated with the activation of IRF1, which is crucial for the upregulation of PD-L1 [57]. Indeed, high levels of nuclear IRF1 in melanoma cells correlate with better PFS in patients treated with anti-PD-1 therapy [58]. In addition, the primary response to anti-PD-1 therapy was associated with and predicted by preexisting IFN-γ and IFN-γ-related signatures, including the expression of *MHCII* in melanoma cells [59]. A subset of patients with melanomas expressing *MHCII* under basal or IFN-γ conditions presented increased PFS and OS, as did the frequency of tumor-infiltrating T-cells in response to anti-PD-1 treatment [60]. In turn, the primary response to anti-CTLA-4 therapy requires robust melanoma *MHCI* expression [59]. IFN-γ increases the levels of the immunoproteasome subunits PSMB8 and PSMB9, whereas increased expression of these genes stimulates anti-melanoma IFN-γ production by tumor-infiltrating lymphocytes, which is associated with an enhanced response to ICIs [61]. The T-cell-induced IFN-γ gene signature accompanying the downregulation of wingless-type (WNT) and MYC signaling in melanoma is associated with a better response of melanoma patients to ICIs [51]. The inhibition of MET [62] and JAK/STAT/IRF1 signaling by 15-deoxy-delta^12,14^-prostaglandin J2 [63] was sufficient to inhibit the IFN-γ-dependent upregulation of PD-L1 in melanoma cells [62], indicating the importance of other regulators of IFN-γ-dependent immune checkpoints in melanoma cells. In addition, IFN-γ increased *CTLA4* expression via a canonical pathway involving JAK1/2-STAT1. Phosphorylated STAT1 binds to a specific site on the *CTLA4* promoter and modulates CREB-binding protein (CBP)/p300-mediated histone acetylation [64]. However, baseline CTLA-4 levels are also regulated by overactivated BRAF/MEK/ERK signaling regardless of the IFN-γ pathway [64]. In turn, inhibition of mutated BRAF was followed by decreased IFN-γ-induced PD-L1 levels [65]. The loss-of-function mutations in *JAK1* and *JAK2* were associated with the inhibition of the expression of IFN-γ-dependent genes, and this effect could be replicated by the use of a *JAK2* inhibitor in IFN-γ-responsive cells. Notably, exposure of melanoma cells to IFN-γ resulted in the activation of the transcription of genes associated with the downregulation of JAK/STAT signaling, including *SOSC1* [57]. These studies demonstrate that IFN-γ (1) regulates the expression of targets for ICIs via the canonical JAK/STAT-dependent pathway and (2) triggers negative feedback signaling, which can prevent the development of resistance to ICIs, as elevated expression of PD-L1 on the melanoma cell membrane, for example, can be associated with a worse immune response in patients treated with ICIs [66], as discussed later. IFN-γ was shown to upregulate gamma-interferon-inducible lysosomal thiol reductase (GILT), and high GILT levels were associated with improved survival in ICI-treated melanoma patients [67]. In melanoma patients with high expression of IFN-γ-related genes, increased expression of the senescent cell signature was detected, which corresponded to a better response to anti-PD-1 therapy [68]. It has also been shown that immune control of melanoma during the ICI response requires IFN-γ-dependent upregulation of p21, a marker of senescence induction [69]. The response to ICIs is also dependent on the production of chemokines by melanoma cells. In this respect, antagonists of the Toll-like receptors TLR2 and TLR6 synergistically with IFN-γ increase the expression of C-X-C motif chemokine ligand 10 (CXCL10), CXCL11, IL-8, IL-32, and cathepsin S in melanoma cells [70].

### IFN-γ as a biomarker for the clinical response of melanoma patients to ICIs

There is significant interest in developing robust biomarkers in patients who are more likely to respond to immunotherapies. Considering the many studies showing the beneficial effects of IFN-γ in response to ICIs, the importance of IFN-γ itself and IFN-γ-related gene signatures (IFN-γ scores) as biomarkers or predictors of the clinical outcome of melanoma patients treated with ICIs has been extensively considered [71–73]. This is substantiated by the observations that anti-PD-1 and anti-CTLA-4 monotherapy induced high variation in overall gene expression among longitudinal tumor biopsies collected from patients prior to treatment and during therapy, whereas constant patterns of IFN-γ-related gene expression were found during treatment among responders versus nonresponders [74]. Compared with *IFNG*^low^ patients, melanoma patients treated with pembrolizumab who exhibit high expression of *IFNG* have increased PFS [75]. Basal serum levels of IFN-γ are associated with the disease control rate and OS in melanoma patients treated with ICIs [76]. In a study assessing the serum levels of several cytokines, the levels of IFN-γ, IL-10 and IL-6 were significantly greater in responders than in patients who did not respond to nivolumab [29]. Increased baseline transcript levels of IFN-γ and a greater ratio of IFN-γ/IL-10 were found in samples of peripheral blood from melanoma patients who responded to anti-PD-1 therapy than in those from nonresponders [77]. Patients with increasing IFN-γ levels 6 weeks after the initiation of treatment with anti-PD-1 therapy had longer PFS [78], whereas recurrence-free survival (RFS) was significantly longer for patients with high IFN-γ scores in another study including a median follow-up of 26 months [79]. High expression of IFN-γ-related genes, including *TBX21*, *STAT1*, *IRF1*, and *IFNG*, is associated with better outcomes than anti-PD-1 monotherapy in melanoma patients [80]. Similarly, patients with high basal or induced IFN-γ scores had a better immune response [52], and PD-L1 levels and enrichment of interferon signatures in melanoma samples were correlated with patient response to anti-PD-1 immunotherapy [57]. T-cell immunoreceptor with Ig and ITIM domain (*TIGIT*) methylation is correlated with the IFN‑γ signature and response of melanoma patients to ICIs [81]. Gene expression analysis of biopsies from melanoma patients who exhibited distinct overall responses to anti-PD-1 monotherapy revealed an increase in IFN-γ-related gene expression in responders. Both the IFN-γ-dependent gene signature, which includes *IFNG*, *CXCL9*, *CXCL10*, *STAT1,* major histocompatibility complex class II DR alpha (*HLA-DRA*) and *IDO1*, and the expanded immune gene signature, which includes 18 genes, were significantly correlated with the best overall response (BOR) and PFS [82]. In turn, elevated expression of IFN-γ-related genes was found in both responding and nonresponding patients, suggesting that a higher IFN-γ score alone was not sufficient to predict the clinical response. The ratio of the IFN-γ signature to the immunosuppression signature (IMS) better stratifies patients who benefit from treatment with anti-PD-1 antibodies than the expression of several genes demonstrated and validated in melanoma by others [83, 84]. Moreover, the ratio of the IFN-γ signature score to the IMS identified via analysis of RNA-seq data was also correlated with the outcome of ICI therapy, and a higher IFN-γ signature score/IMS predicted a greater chance of clinical benefit from anti-PD-1 therapy [85]. Specifically, 18 genes were included in the IMS: fibroblast activation protein alpha (*FAP*), platelet-derived growth factor receptor beta (*PDGFRB*), *CD163*, sialic acid-binding Ig-like lectin 1 (*SIGLEC1*), *IL10*, C–C motif chemokine ligand 2 (*CCL2*), *CCL8*, *CCL13*, inhibin subunit beta A (*INHBA*), versican (*VCAN*), *AXL*, Twist family BHLH transcription factor 2 (*TWIST2*), ADAM metallopeptidase domain 12 (*ADAM12*), collagen type VI alpha 3 chain (*COL6A3*), stanniocalcin 1 (*STC1*), ISG15 ubiquitin-like modifier (*ISG15*), branched chain amino acid transaminase 1 (*BCAT1*), and olfactomedin-like 2B (*OLFML2B*) [85]. In another study, a panel of 15 genes involved in immune checkpoint and activation pathways, including *IFNG*, *CXCL9*, *CXCL10*, *CD8A*, perforin 1 (*PRF1*), granzyme B (*GZMB)*, *PD-1*, *PD-L1*, *PD-L2*, *CTLA4*, *CD80*, *CD86*, *LAG3*, *TIM3*, and B and T-lymphocyte-associated (*BTLA*), was identified as a predictor of melanoma patient response to ICIs [86]. Analysis of melanoma tumor biopsies revealed consistent patterns of IFN-γ gene expression in patients responding to ICIs, which was associated with increased expression of antigen presentation pathway genes, particularly HLA class I and class II genes, such as beta-2-microglobulin (*B2M*), transporter 1 ATP-binding cassette subfamily B member (*TAP1*), *TAP2*, NLR family CARD domain-containing 5 (*NLRC5*), and class II major histocompatibility complex transactivator (*CIITA*), which is the master regulator of MHC-II [51]. The signatures of genes related to inflammatory chemokines, activation of T-cells and IFN-γ signaling are predictive of the immune response to ICIs [87]. Higher expression of genes, including IL-2 inducible T cell kinase (*ITK),* 5’-nucleotidase ecto (*NT5E),* integrin subunit alpha *(LITGAL), CD8A,* C-X-C motif chemokine receptor 6 (CXCR6)*, IFNG, CXCL9*, and *LCK*, efficiently differentiated patients who responded to therapy from those with primary resistance to nivolumab [88]. Examining different markers related to IFN-γ and IFN-γ-dependent gene signatures revealed high predictive value in predicting patient response to ICIs in melanoma patients, especially when assessed during therapy [89]. Melanoma patients who respond to anti-CTLA-4 or anti-PD-1 therapy are also characterized by increased immunoproteasome expression associated with increased cytotoxic immune cell infiltration and upregulation of the IFN-γ pathway [61, 90]. This was particularly associated with high expression of *PSMB8* and *PSMB9* [61]. In addition, analysis of gene expression in melanoma biopsies revealed an IFN-γ-related methylation subtype, which included high expression of *PSMB9* and was characterized by increased OS in patients [91]. Patients with mutations in *ARID2*, encoding a subunit of the SWI/SNF chromatin remodeling complex, are characterized by higher levels of IFN-γ and better responses to anti-PD-1 immunotherapy [92]. The basal level of Ly6E^high^ neutrophils, which are induced by the activation of the stimulator of interferon genes (STING) signaling pathway and can sensitize nonresponsive tumors, was identified as a biomarker of the response to anti-PD-1 therapy [93].

The IFN-γ-related signature also had clinical significance in patients treated with the ICI combination. A high IFN-γ-related gene expression signature and high tumor mutational burden (TMB) were associated with a low risk of relapse in a follow-up study on neoadjuvant ipilimumab plus nivolumab trials OpACIN (NCT02437279) and OpACIN-neo (NCT02977052) [94]. Accordingly, the signature of IFN-γ-dependent genes in combination with high TMB among patients treated with PD-1 monotherapy and combination therapy with anti-PD-1 and anti-CTLA-4 correlated with better PFS and OS values in responding patients [95]. The coexpression of nine genes including *CCL5*, guanylate-binding protein 5 *(GBP5)*, *GZMA*, *GZMH*, *IRF1*, *LAG3*, natural killer cell granule protein 7 (*NKG7)*, *PRF1*, and proteasome 20 s subunit beta 10 (*PSB10)* was correlated with the infiltration of CD8 + T-cells and was associated with the response to IFN-γ, antigen presentation and processing. Notably, this nine-gene signature was validated via publicly available datasets, which included follow-up data from ICI-treated patients, also patients with melanoma. The gene signature could significantly differentiate responders from patients with progressive disease and low co-expression of these genes was related to shorter survival in patients treated with ICIs. Consequently, this gene signature is considered a biomarker for both the survival of melanoma patients and the response to ICIs [96].

### Role of IFN-γ in the resistance of melanoma patients to ICIs

#### Types of resistance to ICIs

**Fig. 4:**
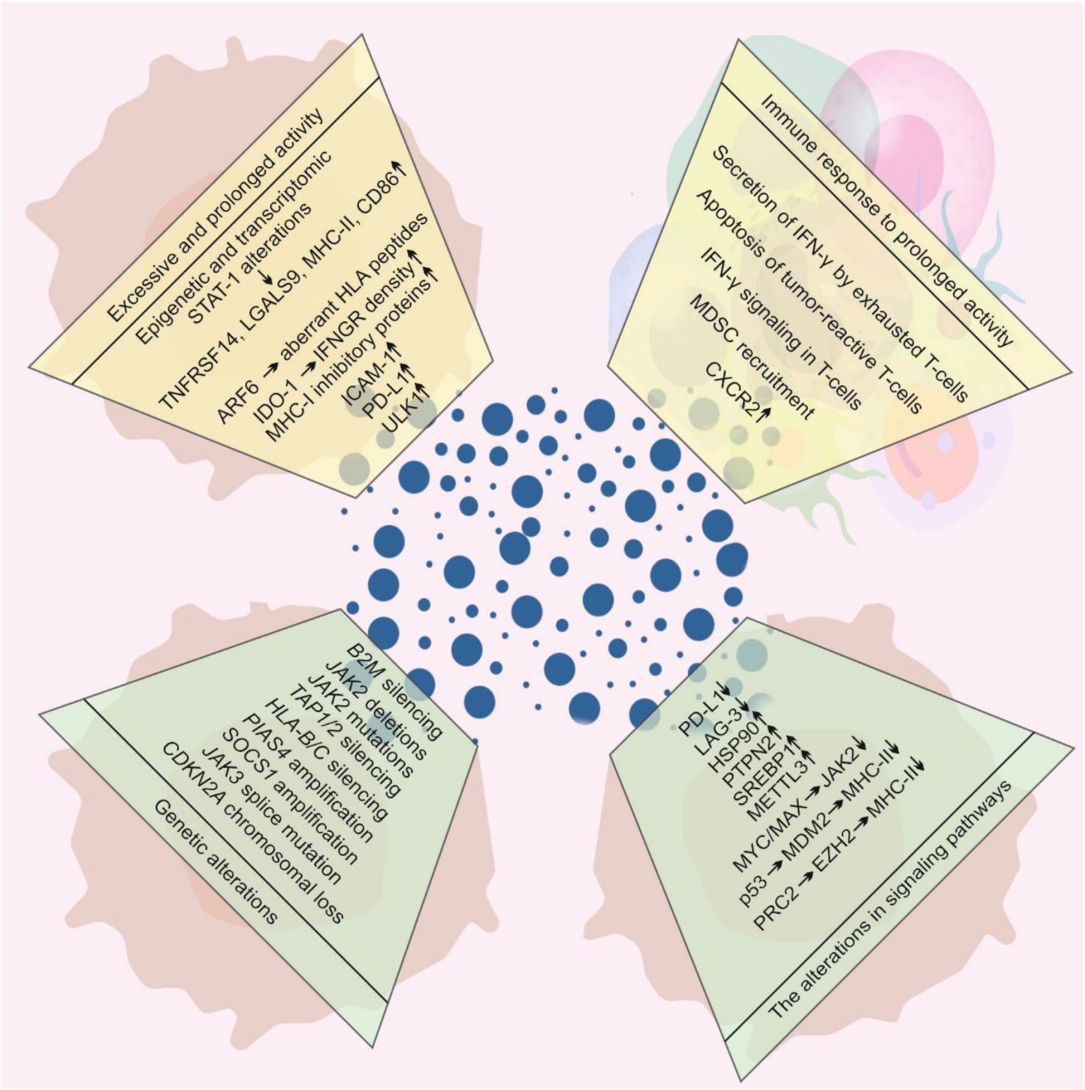
The mechanisms of IFN-γ-related resistance of melanoma patients to ICIs. Resistance to ICIs can develop owing to prolonged exposure of immune cells and melanoma cells to IFN-γ, leading to overactivation of IFN-γ-induced signaling pathways and altered expression of IFN-γ-regulated genes (yellow background). Conversely, the loss of cell responsiveness to IFN-γ (green background) can be associated with either genetic alterations in the components of the JAK/STAT cascade or other changes in intracellular signaling pathways that restrict the effective activation of beneficial IFN-γ-mediated response. A detailed description of these mechanisms is included in the text

The lack of response or resistance of melanoma cells to ICIs is a growing clinical problem. Defining the exact mechanisms of resistance to immunotherapy with ICIs that could clearly explain the lack of response is difficult owing to inter- and intramelanoma heterogeneity [95]. Three major classes of resistance to ICIs can be distinguished: (1) primary resistance, (2) adaptive resistance, and (3) acquired resistance [66]. Primary resistance occurs when patients do not respond to immunotherapy because of tumor-extrinsic features associated with immunodeficiencies or tumor-intrinsic features such as a lack of antigen or insensitivity to T-cell responses, e.g., due to a defective IFN-γ-dependent pathway [66, 97]. Mice bearing *IFNGR1*^KO^ melanoma tumors do not respond to anti-CTLA-4 therapy, indicating that impairment of the IFN-γ signaling pathway is associated with primary resistance to CTLA-4 inhibition [8]. An in vivo CRISPR-Cas9 screening approach in transplantable tumors in mice treated with ICIs confirmed that defects in IFN-γ signaling caused resistance to immunotherapy [98]. Primary resistance to ICIs can largely limit their clinical efficacy, as demonstrated for stage IV melanoma patients with primary resistance to anti-PD-1 therapy, who have 3-year OS rates of 10% compared with 65% for patients who respond to this treatment [99]. During adaptive immune resistance, the patient’s immune system recognizes cancer cells, but cancer cells dynamically adapt to the immunological response, e.g., by upregulating mechanisms that protect them from the T-cell-mediated response. In turn, acquired resistance is characterized by a preliminary response to treatment and activation of the immune system, which is followed by the acquisition of mechanisms leading to tumor relapse and progression [66, 97]. The mechanisms of the contribution of IFN-γ to the response and resistance to ICIs in melanoma patients are summarized in Fig. 4.

#### Role of IFN-γ-regulated signaling in the resistance of melanoma to ICIs

IFN-γ can act as a double-edged sword that strengthens antimelanoma immunity, while leading to the long-term acquisition of resistance to ICIs [100]. The distinct mechanisms of primary and acquired resistance to ICIs may be associated with dysregulation of IFN-γ-dependent signaling either by excessive and prolonged activity of this cytokine or, conversely, by a lack of melanoma cell responsiveness to IFN-γ due to genetic alterations. IFN-γ secretion from CD4 + T-cells was increased in patients who progressed on anti-PD-1 therapy [101]. A strong IFN-γ-dependent signature is associated with a poor response to ICIs in melanoma patients, which is associated with IFN-γ-mediated upregulation of classical and nonclassical MHC-I inhibitory proteins facilitating immune escape [102]. Adaptive immune resistance driven by IFN-γ requires ADP ribosylation factor 6 (ARF6) activation, as ARF6 controls the plasma membrane density of IFNGR [103]. NAMPT synergizes with IFN-γ to regulate *PDL1* expression, leading to immune evasion by melanoma cells. This, however, could be exploited for anti-PD-1 immunotherapy, as patients with NAMPT^high^ melanomas are more sensitive to anti-PD-1 therapy [104]. The promotion of high levels of PD-L1 on both melanoma and immune cells by IFN-γ led to the development of resistance to anti-CTLA-4 in combination with radiation [100]. This was associated with prolonged IFN-γ-dependent STAT1-related epigenomic and transcriptomic alterations causing reduced infiltration of immune cells via preferential activation of several genes such as TNF receptor superfamily member 14 (*TNFRSF14),* galectin-9 *(LGALS9), MHCII,* and *CD86* [100]. Mechanistically, removal of IFNGR/IFNAR or pharmacological inhibition of JAK restored the sensitivity of melanoma cells to ICIs [100]. Furthermore, inhibiting tumor IFN-γ signaling might increase the expression of interferon-stimulated genes in immune cells as a result of elevated IFN-γ secretion by exhausted T-cells [105]. In this respect, prolonged IFN-γ signaling can have opposite effects on melanoma cells and immune cells to establish a regulatory relationship that limits both adaptive and innate immune responses. Notably, even transient exposure to IFN-γ can elicit long-term inflammatory responses in melanoma via the “catch and release” mechanism associated with the capture of IFN-γ by phosphatidylserine on the surface of viable melanoma cells [106]. In addition, IFN-γ-activated T-cells contribute to the formation of cell–cell structures, which evade the response of T-cells during exposure to ICIs [107]. In response to anti-PD-1 treatment, CD8 + T-cell-produced IFN-γ induces the Nod-like receptor protein 3 (NLRP3) inflammasome, which is associated with the concurrent release of heat shock protein 70 (HSP70) and the release of WNT-5a. WNT-5a activated the Hippo/Yes-associated protein (YAP) pathway, leading to an increase in the expression of C-X-C motif chemokine receptor 2 (*CXCR2*). CXCR2 regulates the immune escape of melanoma cells by suppressing cytolytic T-cells through the recruitment of granulocytic subsets of myeloid-derived suppressor cells (MDSCs) [108]. Genetic or pharmacological inhibition of NLRP3 reduces the subset of MDSCs, augments antimelanoma immunity, and cooperates with anti-PD-1 antibodies to reduce tumor growth [109]. IFN-γ in the tumor microenvironment might affect the response to a combination of anti-PD-1 and anti-CTLA-4 agents by inducing apoptosis in tumor-reactive T-cells in melanomas with a low mutational burden [110]. Unc-51 like kinase 1 (ULK1) was shown to augment the IFN-γ-induced expression of immunosuppressive genes while sparing the IFN-γ-dependent effects on immunostimulatory genes. Consequently, the inhibition of ULK1 in combination with anti-PD-1 therapy suppressed melanoma growth in vivo [111]. Melanoma patients who do not respond to ICI monotherapy frequently express alternate immune checkpoints, including IDO1 [80]. Prolonged exposure of melanoma cells to IFN-γ was associated with IDO1-mediated depletion of tryptophan followed by the bypass of tryptophan codons by ribosomes in the absence of tryptophan. Consequently, this results in the presentation of aberrant HLA peptides [112]. The combination of anti-CTLA-4 monotherapy with an IDO1 inhibitor resulted in melanoma tumor regression and was dependent on the activity of CD8 + T-cells and IFN-γ [113]. The use of PEGylated kynureninase, which degrades kynurenine into immunologically inert metabolites, in combination with anti-PD-1 therapy increases the survival of mice with melanoma [114]. Interestingly, IFN-γ can also upregulate intercellular adhesion molecule-1 (ICAM-1) and PD-L1 on melanoma-derived exosomes, which contributes to the suppression of the T-cell response [115].

In contrast to prolonged overactivation of the IFN-γ/JAK/STAT signaling pathway, a decrease in or lack of responsiveness to IFN-γ can also lead to ICI resistance due to alterations in the activity of alternative signaling pathways, which restrict responsiveness to IFN-γ. Patients who are nonresponsive to anti-PD-1 antibodies present an immune signature associated with lower levels of IFN-γ-regulated proteins such as PD-L1 and LAG-3, either before or during therapy than do responding patients [74]. Single-cell RNA sequencing revealed reduced expression of IFN-γ-regulated genes as a part of resistance to anti-PD-1 therapy [116]. Combining HSP90 inhibition and anti-CTLA-4 monotherapy efficiently reduced melanoma tumor size, which was associated with an increase in the expression of IFN-γ-regulated genes [117]. Since downregulation of MYC was identified as an ICI-responsive alteration in melanoma cells [51], overexpression of *MYC* in melanoma cells was associated with resistance to anti-PD-1 immunotherapy [118]. Mechanistically, MYC-overexpressing cells exhibit lower responsiveness to IFN-γ because of MYC/MAX-mediated downregulation of *JAK2* expression [118]. Reduced responsiveness to IFN-γ during ICI therapy can be associated with the activity of enhancer of zeste 2 (EZH2), a part of the polycomb repressive complex 2 (PRC2) complex. Inhibition of EZH2 enabled IFN-γ-induced *MHCII* expression by increasing chromatin accessibility at the *CIITA*. In addition, an inverse correlation between PRC2 complex-related gene expression and the response to anti-PD-1 treatment was found in melanoma patients [119]. In addition, the inhibition of mouse double minute 2 (MDM2) followed by p53 activation was associated with the upregulation of genes related to IFN-γ signaling, including *MHCII*, in melanoma [120], which is consistent with the previously reported contribution of p53 to IFN-γ-dependent PD-L1 regulation [121]. Deletion of protein tyrosine phosphatase nonreceptor type 2 (*PTPN2*) increased the efficacy of ICIs in melanoma cells, as PTPN2 diminished the IFN-γ-mediated effects on antigen presentation and suppression of tumor growth [23, 98]. The inhibition of methyltransferase 3, N^6^-adenosine-methyltransferase complex catalytic subunit (METTL3) enhances the response to anti-PD-1 therapy in melanoma by stabilizing the IFN-γ/STAT1/IRF1 pathway [122]. Taken together, these studies show that multiple signaling pathways can contribute to the diminished responsiveness of melanoma cells to IFN-γ, which results in diminished sensitivity to the immune response during ICI therapy.

The mechanisms of attenuated IFN-γ signaling may involve a genetic background associated with the inactivation of distinct components of IFN-γ-dependent signaling. Nonresponders to anti-CTLA-4 therapy are characterized by the loss of several IFN-γ-related genes and the amplification of *SOCS1* and *PIAS4*, which is associated with the inhibition of the IFN-γ pathway [8]. IFN-γ-resistant HLA class I-positive melanoma metastases can evolve into HLA class I-negative lesions characterized by complete insensitivity to CD8 + T-cells due to the coordinated silencing of genes involved in antigen presentation, such as *HLA-B*, *HLA-C*, *B2M*, *TAP1*, and *TAP2*. Most notably, mutations affecting IFN-γ signaling-related genes were also found in primary tumors, although with a substantially lower frequency than in metastases [123]. Both *JAK1* and *JAK2* knockout melanoma cells are insensitive to anti-PD-1 and anti-CTLA-4 therapy [124, 125]. In relapsed tumors from anti-PD-1-treated patients, acquired mutations in *JAK1* and *JAK2* were detected, resulting in a complete lack of *JAK2* expression and downregulation of genes associated with the JAK/STAT signaling pathway [126]. Although melanoma cells can still be recognized by T-cells, mutations in *JAK1*/*2* render them resistant to the effects of IFN-γ [126]. Consequently, cells are not susceptible to the antiproliferative effects of IFN-γ and lack the IFN-γ-induced surface expression of PD-L1 and MHC-I [126, 127]. Surprisingly, melanoma cells lacking IFNGR1 exhibited activated JAK1/2, which was a consequence of increased mTOR activity [128]. A poor responder to a combination of anti-PD-1 and anti-CTLA-4 therapy has a loss-of-function *JAK3* splice mutation, leading to low expression of *JAK3* [95]. Mutations and deletions of *JAK2* are associated with chromosomal loss of the tumor suppressor cyclin-dependent kinase inhibitor 2A (*CDKN2A*), which is associated with resistance to IFN-γ [129]. Resistance associated with JAK1/2 deficiency can be overcome by the coadministration of a TLR9 agonist and anti-PD-1 antibody [125]. Additionally, ICI-resistant melanomas can have point mutations, deletions or loss of heterozygosity (LOH) in *B2M*, thus causing disturbances in the presentation of MHC-I, preventing the identification of melanoma cells by the immune system [130]. Interestingly, the secretion of IFN-γ is significantly lower in *B2M*-knockout melanoma cells cocultured with matched tumor-infiltrating lymphocytes than in control cells [131], although *B2M*-knockout melanoma cells respond to IFN-γ via the upregulation of typical IFN-γ-dependent genes, but are not recognized by antigen-specific T-cells due to a lack of surface MHC-I proteins [125]. Loss of IFN-γ signaling due to genetic perturbations can also contribute to reduced infiltration of CD8 + T-cells [127]. In addition, T_reg_ cells repress IFN-γ derived from CD8 + T-cells to sustain TAMs by promoting sterol regulatory element binding transcription factor 1 (SREBP1)-dependent lipid metabolism, while inhibition of the SREBP1 pathway cooperates with anti-PD-1 therapy to reduce melanoma tumor growth [132].

### Plasticity of the melanoma cell phenotype in response to IFN-γ

**Fig. 5:**
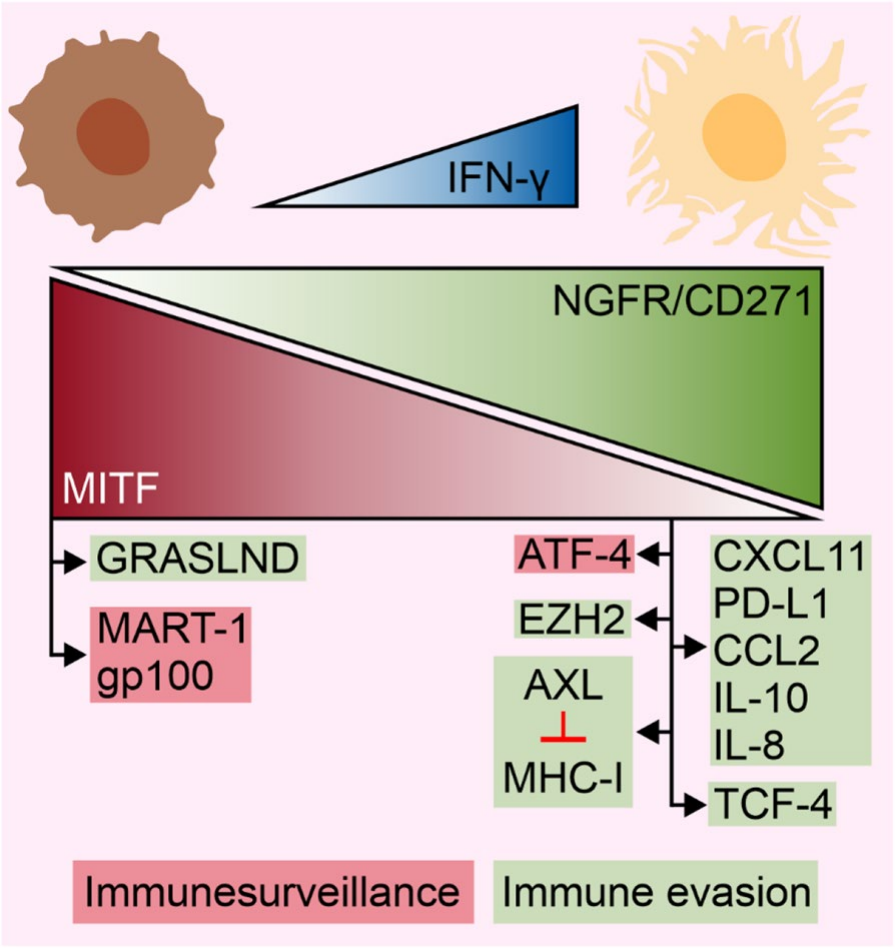
The influence of IFN-γ on the plasticity of melanoma cell phenotype. An increased/prolonged exposure of melanoma cells to IFN-γ leads to the diminution of microphthalmia-associated transcription factor (MITF) level and activity, and an increase in the frequency of nerve growth factor receptor (NGFR)/CD271 cells. Consequently, IFN-γ promotes the switch of melanoma cell characteristics from differentiated phenotype (MITF^high^/NGFR^low^) to neural crest stem-like dedifferentiation phenotype (MITF^low^/NGFR^high^). These boundary phenotypes of melanoma cells are additionally characterized by increased or decreased levels of specific proteins, which contribute to either immune surveillance or immune evasion in melanoma patients treated with ICIs

While the majority of the aforementioned effects of IFN-γ activity have also been reported in other types of cancer, IFN-γ can largely affect the melanoma-specific regulators involved in the determination of a variety of melanoma cell phenotypes that have a direct impact on the susceptibility of melanoma cells to anticancer treatments, including ICIs. IFN-γ was shown to modulate pigmentation and differentiation programs by affecting central regulators of these processes, including microphthalmia-associated transcription factor (MITF) [133]. IFN-γ decreased tyrosinase activity and melanin content in both melanocytes and melanoma cells. Notably, the inhibitory activity of IFN-γ was found under both basal and α-melanocyte stimulating hormone (α-MSH) conditions [134]. In melanocytes, IFN-γ signaling promotes hypopigmentation by arresting melanosome maturation, which is associated with the downregulation of dopachrome tautomerase (DCT) and tyrosinase (TYR) [135]. IFN-γ reduces the expression of *MITF* as a result of the inhibition of CREB binding to the *MITF* promoter via the association of CREB-binding protein (CBP) with STAT1 but not with CREB [134]. In another study, however, IFN-γ induced melanogenesis through its role in the posttranslational modification of tyrosinase, which was presumably independent of MITF activity as downregulating the level of MITF especially during prolonged exposure was reported [136]. Melanoma dedifferentiation, a state characterized by a low level and activity of MITF, among other factors, has been connected to resistance to available therapies [137], whereas distinct trajectories between the IFN-γ and MITF signaling pathways are closely related to the resistance state of melanoma to immunotherapy [138]. Surprisingly, biopsies of melanoma tumors from patients who responded to anti-PD-1 therapy exhibited features of treatment-induced dedifferentiation [139]. In this respect, chronic exposure of melanoma cells to IFN-γ leads to global chromatin landscape alterations, whereas IFN-γ-induced dedifferentiation is correlated with improved outcomes in melanoma patients [139]. The antimelanoma activity of IFN-γ relies on diminished protein translation, which is followed by the activation of the stress response associated with the activating transcription factor-4 (ATF-4)^high^/MITF^low^ phenotype of melanoma cells. MITF downregulation correlated with improved patient outcome in response to ICIs, but this mechanism was prevented by the inhibition of IDO1 [140], indicating complex consequences of IDO1 modulation in response to ICIs. Conversely, downregulating MITF can also contribute to the modulation of the melanoma cell phenotype with an affected immune response [141], although immune evasion by MITF^high^ melanoma cells has been recently reported to be mediated by glycosaminoglycan regulatory associated long noncoding RNA (GRASLND) [142]. MITF levels are inversely correlated with transcription factor 4 (TCF4) [143] and EZH2 [144], which promote the mesenchymal-like state of melanoma associated with the suppression of antigen presentation. The dedifferentiated phenotype (MITF^low^/AXL^high^) is associated with decreased expression of *MHCI* and resistance to anti-PD-1 therapy [131]. IFN-γ can potentiate immunosuppression via the upregulation of nerve growth factor receptor (NGFR)/CD271 and the downregulation of melanoma antigen recognized by T-cell-1 (MART-1) and premelanosome protein (PMEL/gp100) [145], thereby promoting the MITF^low^/NGFR^high^ neural crest stem-like dedifferentiation phenotype of melanoma cells. NGFR^high^/PD-L1^high^ melanoma cells exhibit vasculogenic mimicry in proximity to immune cells, while distinct NGFR spatial patterns are found depending on tumor localization [146]. Compared with differentiated cells, dedifferentiated melanoma cell lines respond to IFN-γ with increased inflammatory signaling, which is accompanied by enhanced secretion of cytokines and increased PD-L1 levels resulting from increased IRF1 activity [141]. Overall, IFN-γ can modulate the differentiation plasticity of melanoma cells (Fig. 5), which can create potential therapeutic vulnerability to induce nonapoptotic cell death pathways in melanoma [147], including ferroptosis [148]. Mechanistically, T-cell secreted IFN-γ inhibits the expression of solute carrier family 7 member 11 (*SLC7A11*) in melanoma cells, leading to reduced cell viability, which can be further potentiated by ferroptosis-inducing agents [149–151].

## Conclusions and future perspectives

This review discusses the role of IFN-γ as a crucial factor in melanoma biology, with a particular focus on both improving patient response to ICI therapy and developing resistance to this type of treatment. IFN-γ exhibits pleiotropic activity [152] by modulating the activity of the immune system and melanoma signaling pathways, thus, it can strongly affect the therapeutic response of melanoma patients. The baseline level of IFN-γ is one of the factors associated with the response to ICIs, while additional analysis of IFN-γ-dependent gene expression indicated dynamic changes in IFN-γ-regulated signaling early during treatment and after the acquisition of resistance. Unfortunately, current biomarkers still lack the power to stratify melanoma patients accurately. Since clinical trials testing neoadjuvant anti-PD-1 monotherapy in melanoma patients have shown response rates of only 30–55% [84, 153], and combinations of ICIs are frequently associated with substantial adverse effects [154–156], considering the IFN-γ signature as an effective biomarker for patient selection is highly important, while simultaneously indicating that screening for mechanisms of IFN-γ resistance should be considered when therapeutic decisions are made. In this respect, a more comprehensive classification of melanoma at the genetic level and a better understanding of melanoma immunooncobiology can provide evidence of crucial IFN-γ response elements with the potential to predict an effective response to ICI therapy, e.g., by using multimodal integrative platforms focused concomitantly on tumor cells and the tumor microenvironment [146, 157–162]. This is exemplified by a recent study in which spatial proteomics was used to determine the role of sirtuin 1 (SIRT1) as a regulator of T-cell infiltration in melanoma, while pharmacological SIRT1 activation increased the response to anti-PD-1, which was accompanied by increased levels of IFN-γ [163].

The nuanced effects of IFN-γ activity can be the most challenging problem in the appropriate evaluation of its influence on melanoma and immune cells. A structure-based approach to decoupling IFN-γ pleiotropy provided interesting observations that (i) various downstream effectors may exhibit different thresholds for activation by IFN-γ and that (ii) partial (biased) agonists can be used to uncouple the expression of IFN-γ-dependent genes, as exemplified by the expression of *MHCI* and *PDL1*, enabling the presentation of tumor antigens without concomitant immunosuppression associated with the expression of immune checkpoint genes [2]. This view is further complicated by recent comprehensive studies showing that IFN-γ can diffuse to modulate the melanoma tumor microenvironment via bystander effects [164] and that the spread of IFN-γ within melanoma tumors is governed by a diffusion-consumption model, whereas the inequality in IFN-γ exposure of distinct cells within the tumor mass can create, e.g., variability in melanoma cell susceptibility to T-cells, leading to immune evasion of specific melanoma cells [165]. Melanoma heterogeneity may largely affect the influence of IFN-γ and contribute to the development of resistance to ICIs [166] and investigating the contribution of IFN-γ to the response of cancer patients to next-generation immunotherapies is crucial [167–170]. In this respect, the increasing number of small-molecule inhibitors of JAK/STAT signaling may be considered a part of the adjuvant approach for ICIs [171, 172], although the dual role of IFN-γ associated with the activity of the JAK/STAT pathway in the response of melanoma patients to ICIs might limit the clinical efficacy of this approach. Similarly, clinical trials combining the coadministration of ICIs with IFN-γ, as reported in a recent phase 1 study of nivolumab and IFN-γ for patients with advanced solid tumors [173], should be performed with caution because bringing clinical benefit to the majority of melanoma patients treated with ICIs would be impossible without elucidating the specific mechanisms or primary, adaptive and acquired resistance [174–178]. Furthermore, as a greater frequency of IFN-γ-positive memory T-cells exhibiting superior antitumor efficacy has been recently shown in a mouse melanoma model after treatment with anti-CTLA-4 than after anti-PD-1 therapy [179], the role of IFN-γ in compensatory mechanisms triggered after the inhibition of a single immune checkpoint and involving the upregulation of other checkpoint proteins needs to be delineated [31]. This is supported by the observations that patients who progressed on anti-PD-1 therapy exhibited clinical benefit from treatment with ipilimumab administered alone or in combination with nivolumab [101], whereas prior anti-CTLA-4 therapy drove substantial alterations in the tumor microenvironment that affected the subsequent response to anti-PD-1 therapy [180]. Finally, as melanoma cell plasticity is a major clinical problem [181–183], which has consequences for immune escape associated with the contribution of MITF to immunity [184, 185], considering the influence of IFN-γ on the phenotype of melanoma cells is essential during investigations of novel immunotherapy strategies [186].

## Data Availability

No datasets were generated or analysed during the current study.

## Abbreviations

α-MSH: α-Melanocyte stimulating hormone
ADAM12: ADAM metallopeptidase domain 12
APC: Antigen-presenting cells
ARF6: ADP ribosylation factor 6
ATF-4: Activating transcription factor-4
B2M: Beta-2-microglobulin
BCAT1: Branched chain amino acid transaminase 1
BCL-2: B-cell leukemia/lymphoma-2
BOR: Best overall response
BTLA: B and T-lymphocyte-associated
CBP: CREB-binding protein
CBP: CREB-binding protein
CCL13: C-C motif chemokine ligand 13
CCL2: C-C motif chemokine ligand 2
CCL8: C-C motif chemokine ligand 8
CDKN2A: Cyclin-dependent kinase inhibitor 2A
CIITA: Class II major histocompatibility complex transactivator
COL6A3: Collagen type VI alpha 3 chain
CTLA-4: Cytotoxic T-lymphocyte-associated protein-4
CXCL10: C-X-C motif chemokine ligand 10
CXCL11: C-X-C motif chemokine ligand 11
CXCR2: C-X-C motif chemokine receptor 2
CXCR6: C-X-C motif chemokine receptor 6
DC: Dendritic cell
DCT: Dopachrome tautomerase
DR5: Death receptor 5
ERK: Signal-regulated kinase
EZH2: Enhancer of zeste 2
FAP: Fibroblast activation protein alpha
GAS: Interferon-gamma activated site
GBP5: Guanylate-binding protein 5
GILT: Gamma-interferon-inducible lysosomal thiol reductase
GRASLND: Glycosaminoglycan regulatory associated long noncoding RNA
GZMB: Granzyme B
HLA-DRA: Major histocompatibility complex class II DR alpha
HSP70: Heat shock protein 70
ICAM-1: Intercellular adhesion molecule-1
ICI: Immune checkpoint inhibitor
IDO1: Indoleamine 2,3-dioxygenase 1
IFN-γ: Interferon gamma
IFNGR: Interferon-gamma receptor
IL: Interleukin
IMS: Immunosuppression signature
INHBA: Inhibin subunit beta A
IRF1: Interferon regulatory factor 1
ISG: IFN-γ-stimulated genes
ISG15: ISG15 ubiquitin-like modifier
ITIM: Immunoreceptor tyrosine-based inhibitory motif
ITK: IL-2 inducible T cell kinase
ITSM: Immunoreceptor tyrosine-based switch motif
JAK1: Janus kinase 1
JAK2: Janus kinase 1
LAG-3: Lymphocyte-activation gene-3
LGALS9: Galectin-9
LITGAL: Integrin subunit alpha
MART-1: Melanoma antigen recognized by T-cells-1
MDM2: Mouse double minute 2
MDSC: Myeloid-derived suppressor cell
METTL3: Methyltransferase 3, N^6^-adenosine-methyltransferase complex catalytic subunit
MHC-I: Major histocompatibility complex-I
MHC-II: Major histocompatibility complex-II
MIF: Macrophage-migration inhibitory factor
MITF: Microphthalmia-associated transcription factor
NAMPT: Nicotinamide phosphoribosyltransferase
NGFR: Nerve growth factor receptor
NK: Natural killer
NKG7: Natural killer cell granule protein 7
NKT: Natural killer T
NLRC5: NLR family CARD domain-containing 5
NLRP3: Nod-like receptor protein 3
nNOS: Nitric oxide synthase
NT5E: 5’-Nucleotidase ecto
OLFML2B: Olfactomedin-like 2B
OS: Overall survival
PAMP: Pathogen-associated molecular pattern
PD-1: Programmed death-1
PD-L1: Programmed death-1 ligand
PD-L2: Programmed death-2 ligand
PDGFRB: Platelet-derived growth factor receptor beta
PFS: Progression-free survival
PIAS: Protein inhibitor of activated STAT
PMAIP1: Phorbol-12-myristate-13-acetate-induced protein 1
PMEL/gp100: Premelanosome protein
PRC2: Polycomb repressive complex 2
PRF1: Perforin 1
PSB10: Proteasome 20 s subunit beta 10
PTPN2: Protein tyrosine phosphatase nonreceptor type 2
RFS: Recurrence-free survival
sCD74: Soluble CD74
SIGLEC1: Sialic acid-binding Ig-like lectin 1
SIRT1: Sirtuin 1
SKP2: S-phase kinase-associated protein 2
SLC7A11: Solute carrier family 7 member 11
SOCS1: Suppressor of cytokine signaling 1
SOCS3: Suppressor of cytokine signaling 3
SREBP1: Sterol regulatory element binding transcription factor 1
STAT1: Signal transducer and activator of transcription 1
STAT3: Signal transducer and activator of transcription 3
STC1: Stanniocalcin
STING: Stimulator of interferon genes
TAM: Tumor-associated macrophage
TAP1: Transporter 1 ATP-binding cassette subfamily B member
TCF4: Transcription factor 4
TCR: T-cell receptor
T_H_1: T-helper 1-cell
TIGIT: T-cell immunoreceptor with Ig and ITIM domain
TIM-3: T-cell immunoglobulin domain and mucin domain‐3
TMB: Tumor mutational burden
TNFRSF14: TNF receptor superfamily member 14
T_reg_: Regulatory T-cell
TWIST2: Twist family BHLH transcription factor 2
TYR: Tyrosinase
ULK1: Unc-51 like kinase 1
USP18: Ubiquitin-specific peptidase 18
VCAN: Versican
WARS: Tryptophan‐tRNA ligase
WNT: Wingless-type
YAP: Yes-associated protein
